# Placental Pathology in Covid-19 Positive Mothers: Preliminary Findings

**DOI:** 10.1177/1093526620925569

**Published:** 2020-05-12

**Authors:** Rebecca N Baergen, Debra S Heller

**Affiliations:** 1 Department of Pathology and Laboratory Medicine, Weill Cornell Medicine, New York, New York; 2 Department of Pathology, Immunology & Laboratory Medicine, Rutgers-New Jersey Medical School, Newark, New Jersey

**Keywords:** villitis, placenta, pathology, Covid-19, pregnancy, thrombosis

## Abstract

This study describes the pathology and clinical information on 20 placentas whose mother tested positive for the novel Coronovirus (2019-nCoV) cases. Ten of the 20 cases showed some evidence of fetal vascular malperfusion or fetal vascular thrombosis. The significance of these findings is unclear and needs further study.

## Introduction

With the recent pandemic of novel coronavirus (2019-nCoV), hospitals can expect an influx of Covid-19 positive patients to labor and delivery. Not surprisingly, little is known about placental findings in such cases, with only 1 report of 3 cases in the world literature.^1^ Findings reported were nonspecific, including variable degrees of increased perivillous fibrin and focal increased syncytial knots. One placenta had massive infarction, and a chorangioma was present in another. Using reverse transcriptase - polymerase chain reaction (RT- PCR), the authors found no evidence of viral nucleic acids in these 3 cases. Recently, a number of Covid-19 positive patients who have delivered newborns have been seen by us. This report catalogs our experience.

## Materials and Methods

Placentas received by the Department of Pathology at Weill Cornell Medical Center and consisted of 20 cases. Weill Cornell Institutional Review Board approval was given. Due to the infectious nature of the tissue, fixation for 48 hours was performed prior to dissection. Typical sections were fixed in formalin, processed into paraffin blocks, and stained with usual Hematoxylin and Eosin stain. Clinical information was retrieved from the electronic medical record or surgical pathology accession sheet, which is given in Table 1. Testing for Covid-19 was not performed on placental tissue. However, all mothers and infants were tested via RT-PCR at Weill Cornell Department of Pathology and Laboratory Medicine.

**Table 1.** Clinical Information.

Case	Maternal Age	GA	G	P	Birthweight (g)	Delivery	History
1	35	39w6d	6	2032	3650	VD	Focal accreta × 2, fever
2	30	38w0d	8	7017	3360	VD	Fever, GBS+
3	29	40w4d	6	5	3400	VD	Nuchal cord
4	40	39w4d	3	2	3720	CS	PPH, Uterine atony
5	26	39w2d	6	2	3050	VD	
6	40	37w0d	7	5	2072	VD	Meconium, SGA
7	19	38w0d	1	0	2390	VD	Pneumonia, acute hypoxia
8	28	40w3d			3820	VD	Sickle cell trait
9	37	39w0d	4	3	2415	CS	Nuchal cord × 1, ITP, Planned repeat CS, SGA
10	26	40w1d	2	1	3799	VD	
11	40	36wod	2	1	2680	CS	Placenta previa, chronic diabetes
12	38	39w0d	15	10		VD	Readmitted for hypoxia/shortness of breath at 3d postpartum
13	28	40w0d	2	1	3800	VD	HTN
14	40	33w2d	1	0		CS	Severe preeclampsia
15	41	40w0d	1	0	4115	VD	Group B Strep screen positive
16	16	32w2d	3	0	3314	VD	Preterm labor
17	36	35w3d	10	9		CS	Twins, severe preeclampsia
18	23	39w5d	2	1	3580	VD	
19	25	38w4d	2	1	3920	VD	Group B Strep screen positive
20	32	37w6d	3	1	3160	VD	Hypothyroidism

Abbreviations: CS, Cesarean section; d, days; G, gravidity; GA, week of gestation; GBS, Group B Streptococcus carrier status; HTN, hypertension; P, parity; ITP, idiopathic thrombocytopenic purpura; PPH, postpartum hemorrhage; SGA, small for gestational age; VD, vaginal delivery; w, weeks.

## Results

All expectant mothers at our institution are tested for Covid-19 even if asymptomatic and all mothers in this study tested positive. Two mothers had a fever on presentation (cases 1 and 2). In case 7, the mother presented with pneumonia and acute hypoxia but was later discharged home. One woman (case 12) was readmitted for hypoxia and shortness of breath 3 days postpartum. No women were admitted to the intensive care unit or intubated. The remaining women were asymptomatic prior to delivery and in the postpartum period. In all cases, the infants had 5-minute Apgars of 8 or 9, were admitted to the well-baby nursery, and discharged home without apparent sequelae. All infants tested negative for Covid-19 by RT-PCR.

**Figure 1.**
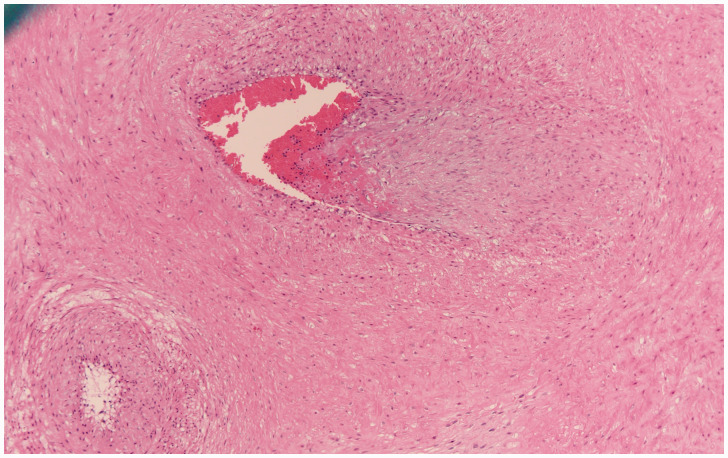
Section of a stem vessel in the placenta showing fetal vascular malperfusion, specifically intramural fibrin deposition where fibrin is deposited in the intima of the vessel. This was the most common type of thrombotic lesion in these placentas. H&E original magnification 200×.

**Figure 2. fig2-1093526620925569:**
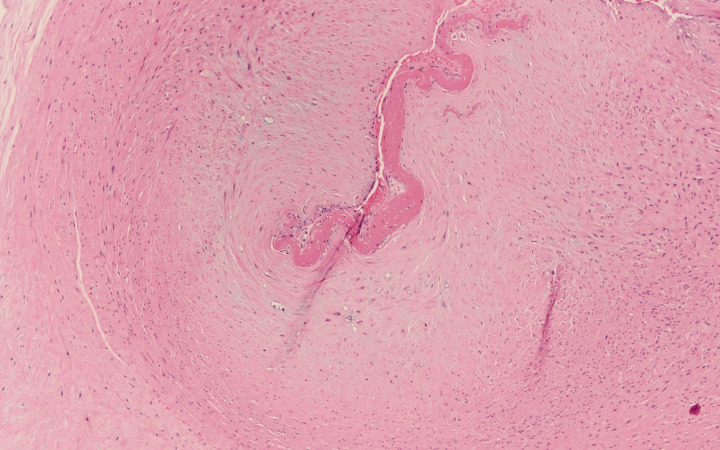
Section of a chorionic plate vessel showing fetal vascular malperfusion, also with deposition of fibrin in the intimal of the vessel extending into the lumen. H&E original magnification 100×.

Table 2 shows a summary of the pathologic diagnoses.^2^ Diagnoses were made and lesions graded as per the Amsterdam criteria.^2^ Interestingly, in these first 20 cases, the most common lesion was fetal vascular malperfusion which was seen in 9 cases (45%). In most cases, this was the presence of intramural fibrin deposition in 1 or 2 foci (cases 2, 12, and 13), 2 cases showed only foci of villous stromal-vascular karyorrhexis (cases 3 and 10), while the remaining cases (1, 4, 5, and 7) showed multiple lesions. A few cases showed intramural nonocclusive thrombi which were very recent. In all cases, the fetal vascular malperfusion was low grade (Figures 1 [Fig fig2-1093526620925569]to 3). Other miscellaneous findings included meconium macrophages (6 cases), lesions of maternal vascular malperfusion (5 cases), and focal increase in perivillous fibrin deposition. One case (7), in which the patient had pneumonia and acute hypoxia, showed evidence of ascending infection with acute chorioamnionitis and acute funisitis. Four cases showed chronic villitis (8, 13, 17, and 18), which was high grade in 2 cases and was associated with obliterative vasculopathy in 1 case (case 8).

**Table 2.** Pathology.

Case	Histology of FVM	Other Findings
1	Thrombosis, intramural fibrin deposition	Focal increase in fibrin, intervillous thrombus, focal chorangiosis, furcate insertion of umbilical cord
2	Intramural fibrin deposition	Meconium
3	Villous stromal-vascular karyorrhexis	Meconium
4	Thrombosis, avascular villi, intramural fibrin deposition	Meconium
5	Thrombosis, intramural fibrin deposition	
6	None	Meconium, maternal vascular malperfusion (infarction, accelerated villous maturity, intraplacental hematoma)
7	Intramural fibrin deposition, avascular villi, villous stromal-vascular karyorrhexis	Decidual vasculopathy, acute chorioamnionitis and funisitis, meconium
8	None	High-grade chronic villitis with associated avascular villi (obliterative vasculopathy)
9	None	Maternal vascular malperfusion (accelerated villous maturity)
10	Villous stromal-vascular karyorrhexis	
11	None	
12	Intramural fibrin deposition	Meconium, early acute funisitis
13	Intramural fibrin deposition	Basal chronic villitis
14	None	Maternal vascular malperfusion (accelerated villous maturity)
15	None	Old retromembranous hematoma, meconium
16	None	Maternal vascular malperfusion (accelerated villous maturity, multiple chorionic cysts)
17	None	Twin 1 – Villous infarctTwin 2 – High-grade chronic villitis
18	None	Low-grade chronic villitis
19	None	
20	None	Hypercoiled umbilical cord, marginal insertion of umbilical cord

Abbreviation: FVM, fetal vascular malperfusion.

**Figure 3.**
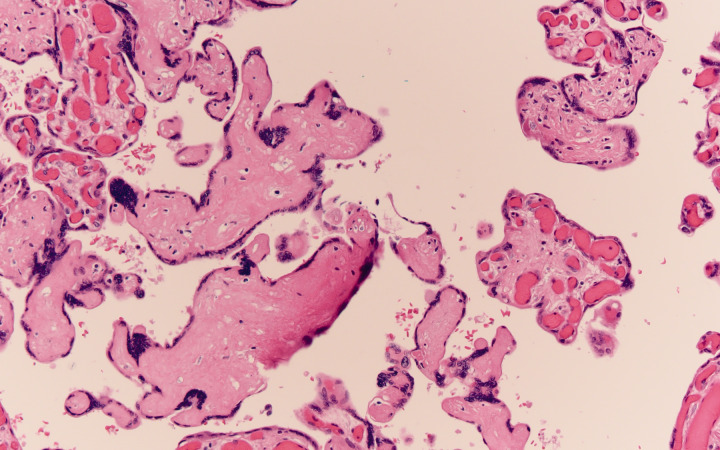
Section of chorionic villi which are avascular. This is another lesion of fetal vascular malperfusion which develops due to thrombosis upstream from the chorionic villi leaving to loss of fetal circulation downstream from the thrombosis. Loss of circulation ultimately leads to loss of fetal vessels with preservation of surface trophoblast. Here, the villi are avascular and the stroma is hyalinized. H&E original magnification 400×.

## Discussion

Very little is currently known about the effects of Covid-19 on the human placenta and neonate. The mouse hepatitis virus, a coronavirus often used as a study model, has been shown to infect the placenta and affect the fetus.^3^ Human SARS has been vertically transmitted and in some cases showed fetal thrombotic vasculopathy (fetal vascular malperfusion).^4^ In humans, early evidence did not demonstrate vertical transmission of Covid-19 in small cohorts of patients.^5,6^ However, demonstration of IgM antibodies to Covid-19 in a single neonate, who also had elevated cytokines suggests that vertical transmission is possible, even if uncommon.^7^

 Covid-19 infection has been associated with hypercoagulability,^8^ with development of ischemic changes including gangrene of fingers and toes, with evidence of d-dimer elevation, and, in some patients, with disseminated intravascular coagulopathy in one series.^9^ Whether the fetal vascular malperfusion in some of the cases described in this study is related to hypercoagulability associated with Covid-19 and whether villitis of unknown etiology is related to an antiviral immune response need further study.

This is a brief report of initial findings seen in placentas of Covid-19 positive mothers. While one of our cases had fetal vascular malperfusion findings potentially related to a furcate cord insertion, in 8 cases there was no gross umbilical cord abnormality known to be associated with fetal vascular malperfusion. This suggests that maternal Covid-19 infection might be associated with propensity for thrombosis in the fetal circulation. This, in turn, may have significant clinical implications for the mother and infant. On the other hand, as the lesions were low grade, more than half did not have thrombotic lesions and the infants tested negative; hence, these findings may be unrelated. Further studies with additional cases are necessary to determine the reproducibility and significance of these initial findings.
